# Adverse childhood experiences as a risk factor for non-suicidal self-injury and suicide attempts in forensic psychiatric patients

**DOI:** 10.1186/s12888-023-04724-w

**Published:** 2023-04-10

**Authors:** Natalie Laporte, Andrejs Ozolins, Sofie Westling, Åsa Westrin, Märta Wallinius

**Affiliations:** 1 Evidence-based forensic psychiatry, Department of Clinical Sciences Lund, Psychiatry, Lund University, Lund, Sweden; 2 Centre for Ethics, Law and Mental Health, Institute of Neuroscience and Physiology, Sahlgrenska Academy, University of Gothenburg, Gothenburg, Sweden; 3 Research Department, Regional Forensic Psychiatric Clinic, Växjö, Sweden; 4 Department of Psychology, Linnaeus University, Växjö, Sweden; 5 Department of Clinical Sciences Malmö, Psychiatry, Lund University, Lund, Sweden; 6 Office for Psychiatry and Habilitation, Psychiatric Clinic Lund, Region Skåne, Sweden; 7 Department of Clinical Sciences Lund, Psychiatry, Lund University, Lund, Sweden; 8 Office for Psychiatry and Habilitation, Psychiatry Research Skåne, Region Skåne, Sweden

**Keywords:** Self-harm, Non-suicidal self-injury, NSSI, Suicide attempt, Forensic psychiatry, Childhood sexual abuse, Childhood physical abuse, Childhood trauma

## Abstract

**Background:**

Exposure to adverse childhood experiences (ACE) have been found to have profound negative consequences on an individuals’ health. Non-suicidal self-injury (NSSI) is a clinically complex and serious global health issue and is closely related to suicide attempts. Previous research has found associations between ACE and NSSI and suicide attempts in clinical samples. However, this association has to our knowledge not been studied to this extent in a sample of forensic psychiatric patients. The aim of this study was therefore to describe the prevalence of adverse childhood experiences (ACE) and their associations with non-suicidal self-injury (NSSI) and/or suicide attempts in forensic psychiatric patients.

**Methods:**

The current study is a cross-sectional study of a consecutive cohort of 98 forensic psychiatric patients (86.7% male) in Sweden. We invited 184 patients with a predicted stay of > 8 weeks who had been cleared for participation by their treating psychiatrist. Of these, 83 declined and 98 eligible patients provided informed consent. Information on ACE, NSSI, and suicide attempts derived from files, self-reports (Childhood Trauma Questionnaire-Short Form; CTQ-SF), and interviews were compared separately among participants with and without NSSI or suicide attempts using *t*-tests. The dose–response association between ACE and NSSI/suicide attempts was analysed using binary logistic regression.

**Results:**

In file reviews, 57.2% of participants reported physical abuse, 20% sexual abuse, and 43% repeated bullying by peers during childhood. NSSI and suicide attempts were associated significantly with CTQ-SF total scores, with medium effect sizes (*d* = .60 to .63, *p* < .01), and strongly with several CTQ-SF subscales. Parental substance abuse was also associated with NSSI (*p* = .006, OR = 3.23; 95% confidence interval [CI] = 1.36 to 7.66) and suicide attempts (*p* = .018, OR = 2.75; 95% CI = 1.18 to 6.42). Each additional ACE factor predicted an increased probability of NSSI (*p* = .016, OR = 1.29; CI = 1.04 to 1.59) but not of suicide attempts. When anxiety and depressive disorders were included in the model, ACE remained a significant predictor of NSSI.

**Conclusions:**

We report extensive ACE, from both files and self-reports. When comparing groups, correlations were found between ACE and NSSI, and ACE and suicide attempts among forensic psychiatric patients. ACE seem to predict NSSI but not suicide attempts in this group, even when controlling for affective and anxiety disorders. Early ACE among forensic psychiatric patients, especially physical and emotional abuse and parental substance abuse, have important impacts on self-harming behaviours that must be acknowledged both by the institutions that meet them as children and in their later assessment and treatment.

## Background

Non-suicidal self-injury (NSSI) is a serious global health issue that includes a range of self-inflicted harmful behaviours such as cutting, burning, self-battering, and hair pulling [1]. NSSI is a complex clinical phenomenon and mainly explained as an emotion regulation coping strategy [2]. A suicide attempt is defined as one, or a set of, actions performed with the expectation that it will lead to one’s death (APA 2013, Sect. 3). While NSSI and suicide attempts can co-exist in one person, the intention of the act determines whether it is defined as either NSSI or a suicide attempt [3]. While suicide attempts are not included in the definition of NSSI, the risk of subsequent suicide attempt has been found to be higher among those who engage in NSSI [4]. Several explanations and functions for NSSI have been presented, often with a focus on identifying risk factors such as dysfunctional emotion regulation, various mental disorders, and adverse childhood experiences (ACE) [5, 6]. The role of ACE may be especially important in the development of NSSI and as a risk factor for attempted suicide [7]. ACE has been associated longitudinally with a wide range of mental disorders [8–11], indicating a possible interaction between ACE, mental disorders, and NSSI and/or suicide attempts.

ACE include a variety of traumatic childhood experiences such as physical, emotional, and sexual abuse; witnessing interparental violence; emotional and physical neglect; parental loss, and maternal separation. Different types of abuse often co-exist and may be related to stressful events in the family [12]. A dose–response pattern has been established between childhood adversities, such as financial strain or other family conflict, and an increased risk of physical, behavioural, mental, and social problems later in life [13]. In a sample of 4859 individuals drawn from a population register, childhood sexual abuse (CSA) was found have a three to five-fold increase of risk for both NSSI and suicidal behaviour [14, 15], and cross-sectional studies based on retrospective data have suggested an association between CSA, psychological strain, and mental health problems such as depression later in life [16, 17]. CSA victims also seem more likely to develop mental disorders [18, 19] and delinquent and violent behaviours [20] than do victims of other forms of abuse and neglect. NSSI and suicide attempts have been strongly associated with CSA in general [21] and specific samples such as juvenile justice-involved youth [22] and female clinical patients [23]. Victims of CSA has been found to be four times more likely to engage in self-harm behavior compared to victims of other types of abuse [21]. Another type of ACE considered important in relation to NSSI and/or suicide attempt is exposure to violence. Childhood exposure to violence is associated with mental health problems later in life [9] including schizophrenia spectrum disorders, affective disorders including major depression, and personality disorders [24]. Exposure to violence as a child has been found to be a strong predictor of suicide in psychiatric populations [25] and in males [24].

Although various ACE have been consistently associated with NSSI and/or attempted suicide, it is important to consider the possible mediation or moderation effects of confounders in such associations. In a sample of incarcerated adults, NSSI and attempted suicide were predicted by CSA in women, but by physical abuse and physical neglect in men [26]. Although a history of childhood abuse (physical, sexual, or emotional) has been associated with NSSI [27], researchers have found this association to be significantly modified when including other factors such as feelings of hopelessness or dissociation [28]. Considering possible mediators seems especially important in heterogeneous patient groups with complex clinical needs.

In clinical forensic psychiatry, patients often present as complex cases with histories of ACE, substance abuse, and other social adversities [29, 30]. The cumulative effect of ACE on psychiatric patients with NSSI and attempted suicides has been understudied [6] and must be considered in more depth.

### Aims

The aim of this study is to describe the prevalence of ACE and its association with NSSI and/or suicide attempts in forensic psychiatric patients, with the following specific objectives:

1. to describe the prevalence of ACE in forensic psychiatric patients; and
2. to explore associations between ACE and NSSI and/or suicide attempts in forensic psychiatric patients to determine group differences in ACE between forensic psychiatric patients with and without NSSI and/or suicide attempts; and
3. to explore dose–response associations between ACE and NSSI and/or suicide attempts, controlling for the impact of mental disorders.

### Design

#### Participants

Patients who met the initial criterion of being cared for at a high security forensic psychiatric clinic in Sweden during the data collection period of November 2016 to November 2020 were candidates for participation. To be included, patients had to have a longer predicted stay than 8 weeks and be able to fulfil the tasks in the study without an interpreter. The sample included only patients sentenced to forensic psychiatric care. Patients under remand or ongoing prison sentences with a temporary need for involuntary psychiatric care were excluded from the study. Prior to participation, all patients were assessed by their treating psychiatrist and were excluded if considered unable to provide informed consent due to acute psychosis or severe intellectual disability or if their participation would worsen their mental health. Individuals who were assessed as unsuitable were excluded from the study. Given the cohort design of the study, availability of participants and previous research within this field [31], a sample of 100 participants was considered appropriate to answer the study research questions. Due to the COVID-19 pandemic, inclusion had to be terminated in November 2020 at a total of 98 participants. Of 277 candidates, 7.9% (*n* = 22) were excluded because of insufficient length of stay, 11.2% (*n* = 31) due to insufficient language skills, and 14.4% (*n* = 40) because of an assessed inability to provide informed consent. Of the remaining 184 eligible patients, 45.1% (*n* = 83) declined participation and 3 patients withdrew their consent before participation, resulting in the study’s final sample of 98 participants (53.3% participation rate). The mean age of the participants was 34.9 years (*range* 19–62, *SD* = 10.7) and 86.7% were male (*n* = 85). Twenty-nine percent (*n* = 28) of the participants had at some point during their lifetime been diagnosed with anxiety disorders and 24.5% (*n* = 24) with depressive disorders. Self-harm (NSSI and/or suicide attempt) was reported among 68.4% (*n* = 67) of the participants, specifically 59% (*n* = 56) had a lifetime history of NSSI and 58.2% (*n* = 57) had a lifetime history of suicide attempt(s). For detailed information on length of stay, previous forensic psychiatric care, and clinical and psychosocial background information in this sample, see Laporte et al. (2021) [32]. During data collection, 9 individuals chose to terminate their participation before all data had been collected, and one self-report was later excluded after being assessed by a senior clinician as non-reliable. Nine of the dropout cases were male and all had different current primary diagnoses and index crimes. Participants had been informed that they could terminate their participation at any time without giving a reason, therefore no causal data for dropout is available. Analyses of self-reports were based on the 88 participants with complete self-reports.

### Procedures

Information on the study was given to all 184 eligible participants by the first author or a fellow PhD student, both with clinical experience with forensic psychiatric patients. After receiving oral and written information on the study, patients who agreed to participate provided written, informed consent. Data was collected through file review and thereafter participants answered self-report questionnaires and, if needed, participated in semi-structured interviews to complement information on suicide attempts, adverse childhood experiences and psychosocial background. If file information was not sufficient and the patient could not provide reliable information regarding an item, this item was coded as missing. If information from files and the patient was contradictive, information from files was used. For self-reports, the data collector was present to provide emotional support or interpret questions if needed. After data collection was completed for each participant, those data were reviewed for quality by the data collector (author NL or data collector JB) and a senior clinician and researcher (author MW) in the field to ensure that data was collected in a similar way between data collectors. No cases were removed from the study after this quality check. Every participant received a small monetary compensation for their contribution to the study.

### Measures

#### NSSI and suicide attempts

Data on lifetime NSSI and on suicide attempts were collected separately in a structured data collection protocol using files (e.g., medical records, forensic psychiatric investigations and assessments, court verdicts) complemented by semi-structured interviews when needed. In this study NSSI is defined as “*the direct, deliberate destruction of one’s own body tissue in the absence of suicidal intent*.” [33]. Participants were asked “*Have you ever deliberately harmed your body without the intention to die*?” with the possible responses “yes”, “no”, “I don’t know” or decline to answer. We defined suicide attempt as a “*nonfatal self-directed potentially injurious behaviour with any intent to die as a result of the behaviour* [, … which] *may or may not result in injury.*” [34]. Participants were asked “*Have you ever made a suicide attempt with the intention to die*?” with the possible responses “yes”, “no”, “I don’t know” or decline to answer. Analyses of NSSI and suicide attempts were based on a total of 95 and 96 responses, respectively. Analyses on NSSI and suicide attempts were performed separately in relation to ACE variables collected by self-reports and files (described below).

#### Adverse childhood experiences

Data on ACE were collected from files, complemented by interviews, and through the self-report measure Childhood Trauma Questionnaire—Short Form (CTQ-SF). The CTQ-SF is a screening tool to detect experiences of childhood abuse and neglect in both adults and adolescents [35]. The CTQ-SF assesses five types of childhood maltreatment over 28 items. Five items in each of the five subscales (sexual abuse, physical abuse, emotional abuse, emotional neglect, and physical neglect) are rated on a 5-point Likert scale (1 = never true; 5 = very often true), and three supplementary items are used to detect minimisation/denial of childhood maltreatment. CTQ-SF subscale scores range from 5 to 25 and provide a quantitative index of the severity of maltreatment experiences for each area. The higher the score, the greater the severity of maltreatment. The following cut-off scores for the five scales are drawn from American samples:

- Emotional abuse: 5–8 = none or minimal; 9–12 = low to moderate; 13–15 = moderate to severe; and 16 +  = severe to extreme.
- Physical abuse: 5–7 = none or minimal; 8–9 = low to moderate; 10–12 = moderate to severe; and 13 +  = severe to extreme.
- Sexual abuse: 5 = none or minimal; 6–7 = low to moderate; 8–12 = moderate to severe; and 13 +  = severe to extreme.
- Emotional neglect: 5–9 = none or minimal; 10–14 = low to moderate; 15–17 = moderate to severe; and 18 +  = severe to extreme.
- Physical neglect: 5–7 = none or minimal; 8–9 = low to moderate; 10–12 = moderate to severe; and 13 +  = severe to extreme [35].

The CTQ-SF can be used as a clinician rating or, as in this study, as a self-report measurement. The Swedish version of the CTQ-SF used in this study demonstrated good internal consistency (α = 0.87), in line with previous studies [36]. The CTQ-SF has proved validity in a number of studies [36, 37] and analyses on CTQ-SF in the current study were based on the 88 participants with complete self-reports after dropouts. If information from files and interviews were inconsistent, the information from files was used.

Information on ACE not covered by the CTQ-SF was collected from files and complemented by interviews. Specifically, information was collected on childhood experiences of parental alcohol and substance abuse, mental illness in parents or other family members, witnessing violence between parents, being exposed to physical or sexual abuse, and parental death(s). These data were categorised as “yes, single occasion,” “yes, multiple occasions,” or “no”. The questions concerning parental alcohol or substance abuse were further categorised as “yes, the mother,” “yes, the father,” “yes, both,” or “no”.

To explore the dose–response effect of ACE on NSSI and suicide attempts, an ACE variable was created from 10 items collected through file reviews and complemented by interviews: experience of being bullied, institutional or foster care placement, parental absence during childhood, parental alcohol abuse, parental substance abuse, parental mental illness, witnessing violence between parents during childhood, and exposure to physical or sexual abuse during childhood. All 10 items included in this ACE variable were dichotomised (0 = no, 1 = yes). Participants could accumulate between 0–10 categories on this variable, i.e. participants with similar scores might have answered yes to different statements but to the same number of statements. Internal consistency for the computed ACE variable had an acceptable Cronbach’s alpha of 0.73.

Accordingly, variables on ACE were analyzed in the following segments: Self-reported data through the CTQ-SF, information on ACE collected through file information and when needed complemented by interview, and through a cumulative ACE scale created from the ACE variable previously described.

### Mental disorders

Information on lifetime diagnoses of mental disorders according to the Diagnostic and Statistical Manual of Mental Disorders, 5th edition (DSM-5) was collected from files (medical records, forensic psychiatric assessments). In the files, diagnoses specified using DSM-IV or ICD-9 formats were converted to DSM-5 by a senior clinician with considerable research experience in the field.

### Psychosocial background

Sociodemographic information (age, gender) and information on psychosocial background (childhood circumstances including socioeconomic status, institutionalization, and foster care placement) was obtained from files complemented by interviews with the participant when necessary.

### Statistical methods

The IBM SPSS Statistics 27 software was used to perform the initial descriptive analyses and Jamovi software to perform tests of bivariate associations (*t*-tests, chi-square tests). For the first aim, descriptive tables were used to report prevalence rates of ACE as reported in files and interviews and interpreted from CTQ-SF scores. Because Levene’s test homogeneity of variance indicated a violation of assumptions of normality in terms of variance, Welch’s *t*-test was used to perform independent samples *t*-tests for the second aim. All bivariate analyses for the second aim were performed using NSSI or suicide attempt as the dependent variable. All categorised ACE variables were dichotomised into “yes” or “no.” In three cases, participants had failed to answer one item each in the CTQ-SF. For those individuals, a mean score for the specific subscale was individually imputed and used in the analyses. For the third aim, binary logistic regression in two models, simple and multiple, was performed. The multiple model included mood disorders and anxiety disorders, based on their possible impact on the model as derived from previous studies. The predictors were screened for multicollinearity with acceptable variance inflation factor values and tolerance. All bivariate analyses were performed using ACE as the dependent variable. Cohen’s *d* was used to present effect sizes.

### Ethical considerations

The senior treating forensic psychiatrist was consulted before any patients were informed of the study, and patients considered as not currently suitable for the study due to psychiatric status or inability to provide informed consent were excluded. All patients who agreed to participate gave informed, written consent before participation. The study, including the monetary reward (which was low in order not to give an incentive that would compromise free consent), was approved by the research ethics committee at Linköping University, Dnr 2016/213–31 and 2017/252–32.

## Results

### ACE in forensic psychiatric patients

In a file review of childhood trauma in the participants, physical abuse was reported for 56 (57.2%) of the participants, on multiple occasions for 48 (49.0%) of these. Repeated CSA was reported for 18 (20.0%) of the participants, and a single occasion of CSA for another 7 (7.8%). Repeated episodes of witnessing interparental violence was reported for 34 (36.2%) of participants, and a single episode for another 8 (8.5%), while 40 (43.5%) of participants had been bullied repeatedly by childhood peers. Half of the participants (*n* = 47; 50.0%) reported no mental health issues among their parents and a majority reported no parental alcohol abuse (*n* = 57; 60.0%,) or substance abuse (*n* = 78, 82.1%). Mental health issues were reported for 28 (29.8%) of participants’ mothers, for 6 (6.4%) of their fathers (*n* = 6), and for 7 (7.4%) of participants, for both parents. Paternal alcohol abuse was reported for 20 (21.1%), maternal alcohol abuse for 5 (5.3%), and alcohol abuse was reported for both parents during childhood for 10 (10.5%). The distribution between paternal and maternal substance abuse was similar (paternal: 7.4%, *n* = 7; maternal: 5.3%, *n* = 5; both: 1%, *n* = 1). In 13 cases (13.7%), one or both parents had died before the participant reached the age of 18. Parental absence during childhood was fairly common, with 27 (*n* = 27.6%) of participants having one parent absent during the majority of their childhood, and 13 (13.3%) having both parents absent. The composite ACE variable creates an overview of the participants’ number of ACEs without valuing the severeness of the individual experience. Of 98 participants, only 3 (3.1%) had experienced all 10 ACE factors and 11 (11.2%) had not experienced any. A majority of the participants (*n* = 38, 38.8%) had experienced 3 to 5 ACE factors during childhood (*Mdn* = 4).

Self-reported ACE as demonstrated by CTQ-SF total and subscale scores are available in Table 1.

**Table 1:** Self-reported ACE among forensic psychiatric patients according to CTQ-SF

CTQ-SF subscale	*Mean score*	*SD*
Emotional abuse	10.67	5.53
Physical abuse	9.56	4.92
Sexual abuse	7.77	5.30
Emotional neglect	13.15	5.81
Physical neglect	9.11	4.28
Minimisation/denial	.54	.93
Total score	50.82	19.98

### Associations between ACE and NSSI or suicide attempts

Providing information on self-reported ACE, Table 2 reports differences between participants with and without NSSI, and those with and without suicide attempts in CTQ-SF total and subscale scores. On average, participants with a history of NSSI reported higher mean total CTQ-SF scores (*M* = 55.6, *SD* = 20.1) than those with no such history (*M* = 44.2, *SD* = 18). Similarly, participants with a history of suicide attempts reported higher mean total scores on CTQ-SF (*M* = 55.9, *SD* = 19.9) than those without (*M* = 43.9, *SD* = 18.2).

**Table 2:** Group differences in self-reported ACE between forensic psychiatric patients with or without NSSI or suicide attempt

	*p*	Mean difference	Lower	Upper	Effect size^a^
**NSSI**					
Emotional abuse	.001	− 3.77	− 5.98	− 1.559	− 0.726
Physical abuse	.085	− 1.82	− 3.90	0.256	− 0.375
Sexual abuse	.097	− 1.75	− 3.83	0.324	− 0.347
Emotional neglect	.018	− 2.93	− 5.36	− 0.506	− 0.519
Physical neglect	.068	− 1.69	− 3.50	0.126	− 0.400
CTQ-SF total	.006	− 11.46	− 19.59	− 3.326	− 0.600
**Suicide attempts**					
Emotional Abuse	.008	− 3.116	− 5.39	− 0.841	− 0.585
Physical Abuse	.004	− 2.846	− 4.74	− 0.956	− 0.622
Sexual Abuse	.465	− 0.820	− 3.04	1.403	− 0.156
Emotional Neglect	.008	− 3.259	− 5.64	− 0.875	− 0.584
Physical Neglect	.006	− 2.435	− 4.15	− 0.722	− 0.599
CTQ-SF total	.004	− 11.971	− 20.10	− 3.839	− 0.628

^a^Effect sizes by Cohen’s *d*

Table 3 presents group differences in ACE, as reported in files and interviews, between forensic psychiatric patients with or without NSSI and suicide attempts. NSSI and/or suicide attempts were more common in patients demonstrating all types of ACE investigated here than would be expected if no group differences indicated associations between NSSI and/or suicide attempts and ACE. However, only a few group differences were statistically significant, mainly in suicide attempts, and wide confidence intervals were noted. Parental substance abuse demonstrated an approximately threefold increase in risk of both NSSI and suicide attempts in forensic psychiatric patients.

**Table 3:** File-reported adverse childhood experiences in forensic psychiatric patients with or without NSSI and suicide attempts

**ACE**	**NSSI (*n* = 56)**			***X***^***2***^	***p***	***CI***	***OR***
	**No**	**Yes**	**Expected yes-count**				
**Parental alcohol abuse**	10	24	20	2.97	.085	0.891–5.31	2.18
**Parental substance abuse**	12	33	26.5	7.31	.007	1.36–7.66	3.23
**Parental mental health issues**	11	30	24.2	6.03	.014	1.23–7.03	2.94
**Parent(s) absent**	15	24	23	0.18	.668	0.521–2.76	1.20
**Witnessed violence**	13	28	24.2	2.60	.107	0.857–4.67	2.00
**Physically abused**	17	37	31.8	4.74	.030	1.09–5.84	2.52
**Sexually abused**	10	15	14.7	0.015	.901	0.418–2.69	1.06
**Bullied during childhood**	15	34	28.9	4.56	.033	1.07–5.72	1.47
**ACE**	**Suicide attempts (*n* = 57)**			***X***^***2***^	***P***	***CI***	***OR***
	**No**	**Yes**	**Expected yes-count**				
**Parental alcohol abuse**	10	25	20.8	3.32	.069	0.931–5.51	2.27
**Parental substance abuse**	13	33	27.3	5.60	.018	1.18–6.42	2.75
**Parental mental health issues**	13	28	24.3	2.36	.125	0.830–4.49	1.93
**Parent(s) absent**	14	25	23.2	0.609	.435	0.604–3.22	1.40
**Witnessed violence**	14	27	24.3	1.25	.264	0.697–3.71	1.61
**Physically abused**	21	34	32.7	0.319	.572	0.557–2.88	1.27
**Sexually abused**	11	14	14.8	0.160	.690	0.330–2.08	0.829
**Bullied during childhood**	15	34	29.1	4.16	.041	1.03–5.45	2.37

In a predictive model, each additional ACE factor predicted an increase in the probability of NSSI (*OR* = 1.29, *p* = 0.016) in a simple model. When including lifetime occurrence of anxiety and depressive disorders in the model, ACE remained a significant predictor while anxiety disorders were borderline predictive of NSSI. For suicide attempts, the ACE variable did not predict increased probability of suicide attempts to a statistically significant degree. When lifetime occurrence of anxiety and depressive disorders was included in the model, only anxiety disorders could significantly predict suicide attempts (specific results are presented in Table 4).

**Table 4:** Cumulative effect of adverse childhood experiences in the prediction of NSSI and suicide attempts in forensic psychiatric patients

**NSSI (*n* = 56)**					**95% CI**		
	**Predictor**	***OR***	***p***	***Z***	**Lower**	**Upper**	***SE***
**Model 1**	Intercept	0.520	.201	− 1.28	0.191	1.42	0.511
	ACE	1.292	.016	2.41	1.049	1.59	0.106
**Model 2**	Intercept	0.346	.065	− 1.847	0.112	1.07	0.575
	ACE	1.291	.026	2.230	1.031	1.62	0.115
	Anxiety	3.496	.055	1.916	0.971	12.58	0.653
	Depression	1.736	.393	0.854	0.490	6.15	0.645
**Suicide attempt (*n*** **= 57)**					**95% *CI***		
	**Predictor**	**Odds ratio**	***p***	***Z***	**Lower**	**Upper**	***SE***
**Model 1**	Intercept	0.834	.714	− 0.366	0.316	2.20	0.495
	ACE	1.178	.103	1.628	0.967	1.43	0.101
**Model 2**	Intercept	0.549	.284	− 1.07	0.183	1.64	0.560
	ACE	1.163	.174	1.36	0.936	1.45	0.111
	Anxiety	4.478	.032	2.14	1.134	17.68	0.701
	Depression	2.086	.270	1.10	0.565	7.70	0.666

The predictive performance of the NSSI model had an accuracy of 0.702, sensitivity = 0.56, specificity = 0.79 (*PPV* = 0.37, *NPV* = 0.66), with an *r*^*2*^ = 0.20 and *p* = 0.033 for the controlled model. For the suicide attempt model, accuracy = 0.62, sensitivity = 0.83, specificity = 0.26 (*PPV* = 0.375, *NPV* = 0.66) were the corresponding values, with an *r*^*2*^ = 0.18 and *p* = 0.008 for the controlled model. Data on the simple model available upon request.

## Discussion

This study reports extensive ACE among forensic psychiatric patients, with only 5 (5.7%) of the participants reporting no emotional abuse during childhood. Furthermore, many participants reported multiple occasions of physical abuse, bullying, and witnessing interparental violence, while CSA was less prevalent but still present. Findings also demonstrated a statistically significant difference between participants with and without NSSI in CTQ-SF total scores and the subscales emotional abuse and emotional neglect. Participants with NSSI or suicide attempts differed significantly in all self-reported ACE variables with medium effect sizes, and parental substance abuse demonstrated a threefold increase in risk for both NSSI and suicide attempts. In the logistic regression, each additional ACE factor predicted an increase of NSSI, and when including lifetime prevalence of anxiety and depression in the model, ACE remained a significant predictor accompanied by anxiety, which increased the risk of NSSI by 3.5 times. ACE itself could not predict suicide attempts, and when including lifetime prevalence of anxiety disorders and depression in the model, only anxiety disorders could predict suicide attempts, but with a 4.5 increase in risk.

### ACE in forensic psychiatric patients

The findings in this study indicate that the absolute majority of this sample of forensic psychiatric patients have endured various forms of ACE. It is evident that the participants in this study grew up under difficult circumstances involving physical abuse, sometimes sexual abuse, interparental physical violence, and bullying by peers. Maternal mental illness and paternal alcohol abuse were also experienced in childhood by this sample. When examining the cumulative ACE factor, we found the majority of participants had experienced 3 to 5 separate ACE factors. Although we did not test for an association between mental disorders and the cumulative ACE effect, previous research has made the connection between the two [38]. Many researchers [39] discuss the role of traumatic events during childhood as a risk factor for impulsivity and delinquency in adulthood; for example, childhood trauma combined with insecure attachment has been found to predict psychological distress and violence in a sample of forensic patients [40]. There is evidence that childhood trauma and different forms of early neglect could cause neurobiological effects that may obstruct the brain’s ability to control and regulate emotions, which might make these individuals more susceptible to impulsivity [41]. This could be especially important to investigate in groups such as forensic psychiatric patients who have complex comorbidities and impulsive behaviours, since initial findings report disinhibition to be associated with reduced neural efficiency during later outcome monitoring or behaviour evaluations [42]. Furthermore, an extensive review concluded that psychological, emotional, social, behavioural, and academic problems in childhood were more common among individuals who had witnessed interparental violence than in those who had not. Witnesses of interparental violence did not, however, differ from those who had experienced physical abuse in terms of the abovementioned childhood problems [43]. It is therefore serious that as many as 42.7% of our sample had witnessed interparental violence during their childhoods. This experience alone might contribute to mental illness and negative development and should be further investigated in this vulnerable population.

Regarding self-reported ACE, we report CTQ-SF scores that are in line with previous reports in comparable samples, such as a clinical sample with comorbid psychiatric and substance use disorders under compulsory care [37] and a sample of psychiatric patients from a high security hospital [35, 37]. All reported scores in the present study were within the low–moderate range for all subscales. In general, participants in retrospective studies of ACE tend to underreport the prevalence of abuse [39], and using self-reports in this area is therefore usually considered a limitation [44]. However, in this study we observed the opposite phenomenon. Information gathered from files presented a lower prevalence ACE than participants self-reported on the CTQ-SF. This is an important finding that might indicate that file information may be insufficient when studying vulnerabilities such as ACE and should therefore be complemented by self-reports.

### Associations between ACE and NSSI or suicide attempts

The findings in CTQ-SF between participants with and without NSSI, and between participants with and without suicide attempts is in line with other studies where emotional and sexual abuse and emotional neglect have been correlated with suicide risk, even after adjusting for sociodemographic and clinical factors [45, 46]. By far the most common self-reported ACE were emotional abuse and emotional neglect, which were the only ones to show significant correlations with both NSSI and attempted suicide. In our clinical experience, these forms of abuse and neglect are seldom inquired for and are therefore rarely, if ever, found in medical records within forensic psychiatry. Emotional maltreatment is, however, a well-studied area [47]. In the context of childhood, emotional maltreatment is the failure of caregivers to provide an environment that is safe, developmentally appropriate, and supportive enough for the child to develop a stable and complete range of emotional and social competencies [48]. Emotional maltreatment has been associated with various depressogenic inferential styles, which are more than just an increased vulnerability to depression [49]. Over time a child subjected to emotional maltreatment is highly likely to develop negative beliefs about themselves and the world because their environment is less predictable than needed, yet its influence is chronic and widespread. Therefore, emotional maltreatment should not be considered less important than physical and sexual abuse; it is not only the most common form of ACE, but also suspected to have a permanent impact on a child’s development [47].

Self-harming behaviours (NSSI and suicide attempts) have been associated with growing up in invalidating home environments, where negative emotions are not accepted and children are often punished for communicating their private experiences and expected to control their emotions and expressions [50]. Growing up with parents who tend to respond to sadness with punishment and/or neglect has been associated, both directly and indirectly, with increased levels of deliberate self-harming [5]. Linehan argued that an invalidating environment, including CSA, deprivation of parental attention, or punishment for the display of emotions, contributes to emotion dysregulation, which in turn contributes strongly to developing self-harming behaviour [51]. This is another reason that emotional maltreatment should be investigated and reported within mental healthcare. An extensive review [45] found a modest but significant association between the CSA and NSSI, but our study sample allowed no such conclusions to be drawn, possibly due to the relatively small sample size. Brown and colleagues [52] found that emotional neglect and abuse seem to be an important factor in the development and maintenance of NSSI, while sexual abuse and physical neglect were mediated by depression and anxiety [52]. This is consistent with the findings in our study, where ACE seems to predict both NSSI and suicide attempts. However, the dose–response relationship between ACE factors and NSSI in this study indicates that although other variables are more important predictors of NSSI in this sample, increased exposure to ACE are linked to NSSI, with an explained variance in the model of 19%. A similar effect was found for suicide attempts, with an 18% explained variance in the model. These findings are supported by findings from a large cohort in a community sample [53], who reported a 31.1-fold risk of ever attempting suicide among individuals with 7 or more ACE factors, and a 2 to 5 times higher risk of suicide attempt in individuals with just one ACE factor in any category. Due to the lack of knowledge regarding adverse childhood experiences within the forensic psychiatric population in general, the study was designed as explorative and no *a prioi* hypotheses were created. In the future, confirmatory studies on the subject are essential and replications of these analyses in different forensic psychiatric samples are needed.

## Conclusions

The present study demonstrates that ACE were highly prevalent in this cohort of forensic psychiatric patients and were associated with self-harm. Using data from the self-report instruments and reviewing file information, we conclude that childhood trauma, especially physical and emotional abuse, is an important concern in this group of forensic psychiatric patients and that its impact on self-harming behaviour in such populations must be acknowledged.

### Strengths and limitations

Difficulties in retrieving scientifically valid and reliable self-report instruments applicable to this population were extensive, and our final decision was based on information from previous studies in similar populations. Since individuals in forensic psychiatry often suffer from cognitive deficits, it was important to choose an instrument with an appropriate level of readability to ensure that the participants understood the questions. However, retrieving information through retrospective studies is difficult, and especially regarding sensitive subjects such as psychological trauma since an individuals’ memory of an event might be altered over time. Also, this study is limited by the regressions being based on relatively small groups which might increase the error margins. It should also be highlighted that this study is based on retrospective data and predictions are not made from a time perspective. However, a major strength of this study was that the data collection relied not only on self-reports, but on a multiple methods design including self-reports, semi-structured interviews, and file reviews. A strength of the study was its cohort design, which ensured that all potential candidates were asked about participation in the study. Given this explorative design no power analysis was performed. This population is an understudied group and the inclusion of all patients regardless of mental disorder and gender increases the generalisability of the current study. Furthermore, the results of the study are not thought to be generalized to the general population, but to other forensic populations such as individuals within the prison and probation services with similarities in demographic and psychosocial background factors and, to some extent, clinical profiles.

## Data Availability

The datasets used during the current study are available from the corresponding author on reasonable request.
